# Long-term follow-up of thoracoscopic ablation in long-standing persistent atrial fibrillation

**DOI:** 10.1093/icvts/ivab355

**Published:** 2021-12-27

**Authors:** Niels Harlaar, Maurice A Oudeman, Serge A Trines, Gijsbert S de Ruiter, Bart J Mertens, Muchtair Khan, Robert J M Klautz, Katja Zeppenfeld, Andrew Tjon, Jerry Braun, Thomas J van Brakel

**Affiliations:** 1 Department of Cardiothoracic Surgery, Leiden University Medical Center, Leiden, Netherlands; 2 Department of Cardiology, Leiden University Medical Center, Leiden, Netherlands; 3 Department of Cardiothoracic Surgery, OLVG, Amsterdam, Netherlands; 4 Department of Cardiology, OLVG, Amsterdam, Netherlands; 5 Department of Biomedical Data Sciences, Leiden University Medical Center, Leiden, Netherlands

**Keywords:** Atrial fibrillation, Long-standing persistent, Thoracoscopy, Ablation, Surgery

## Abstract

**OBJECTIVES:**

Catheter ablation of long-standing persistent atrial fibrillation (LSPAF) remains challenging, with suboptimal success rates obtained following multiple procedures. Thoracoscopic ablation has shown effective at creating transmural lesions around the pulmonary veins and box; however, long-term rhythm follow-up data are lacking. This study aims, for the first time, to assess the long-term outcomes of thoracoscopic pulmonary vein and box ablation in LSPAF.

**METHODS:**

Rhythm follow-up consisted of continuous rhythm monitoring using implanted loop recorders or 24-h Holter recordings. Rhythm status and touch-up interventions were assessed up to 5 years.

**RESULTS:**

Seventy-seven patients with symptomatic LSPAF underwent thoracoscopic ablation in 2 centres. Freedom from atrial arrhythmias at 5 years was 50% following a single thoracoscopic procedure and 68% allowing endocardial touch-up procedures (performed in 21% of patients). The mean atrial fibrillation burden in patients with continuous monitoring was reduced from 100% preoperatively to 0.1% at the end of the blanking period and 8.0% during the second year. Antiarrhythmic drug use decreased from 49.4% preoperative to 12.1% and 14.3% at 2 and 5 years, respectively (*P *< 0.001). Continuous rhythm monitoring resulted in higher recurrence detection rates compared to 24-h Holter monitoring at 2-year follow-up (hazard ratio: 6.5, *P *= 0.003), with comparable recurrence rates at 5-year follow-up.

**CONCLUSIONS:**

Thoracoscopic pulmonary vein and box isolation are effective in long-term restoration of sinus rhythm in LSPAF, especially when complemented by endocardial touch-up procedures, as demonstrated by the 68% freedom rate at 5 years. Continuous rhythm monitoring revealed earlier, but not more numerous documentation of recurrences at 5-year follow-up.

**Figure:**
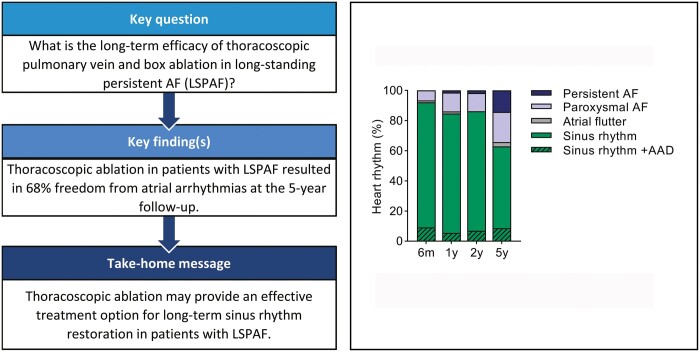

## INTRODUCTION

Long-term rhythm control in long-standing persistent atrial fibrillation (LSPAF) remains challenging to achieve in clinical practice. Catheter ablation has shown modest rates of sinus rhythm in this population, despite repeated procedures [1–4]. In addition to the pulmonary veins (PVs), the left atrial posterior wall has been identified as an important ablation target to isolate the arrhythmogenic substrate driving LSPAF [5, 6]. The creation of transmural long-lasting linear lesions isolating this region, however, has proven difficult through endocardial techniques alone [7]. Epicardial surgical ablation devices have shown to be able to effectively create transmural linear atrial lesions in a minimally invasive setting [8, 9]. Despite totally thoracoscopic ablation becoming increasingly accepted for the treatment of symptomatic (long-standing) persistent atrial fibrillation (AF), long-term follow-up data remain sparse, particularly in the LSPAF population. In this study, we report long-term outcomes of thoracoscopic isolation of the PVs and the left atrial posterior wall in an LSPAF population.

## PATIENTS AND METHODS

### Patient inclusion and data collection

Eighty consecutive patients with symptomatic LSPAF who underwent thoracoscopic ablation at the Leiden University Medical Center (LUMC, 2009–2017) and OLVG (2012–2017) were screened for the inclusion in the long-term rhythm analysis. All patients with a rhythm follow-up beyond the blanking period (>3 months) were included. LSPAF was defined as continuous AF with a duration longer than 12 months [10]. All patients were symptomatic and refractory to 1 or more class I or III antiarrhythmic drugs (AADs) or had failed both AAD and catheter ablation. Clinical data in the electronic patient information system of the Cardiothoracic Surgery/Cardiology departments were retrospectively analysed.

### Surgical procedure

**Figure 1:**
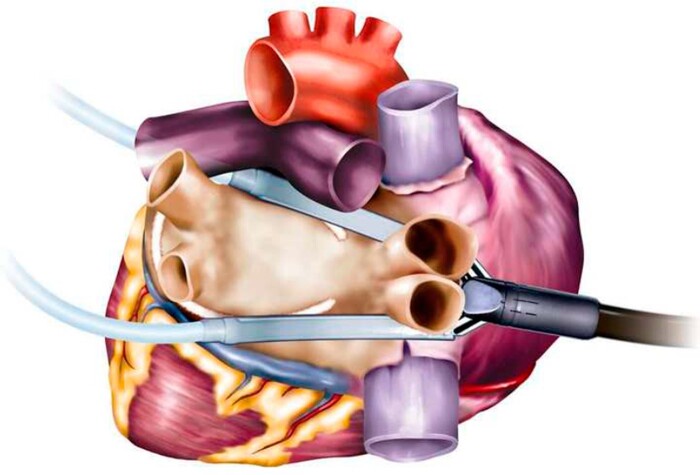
Positioning of the Gemini-S ablation device to isolate the pulmonary veins and left atrial posterior wall.

The thoracoscopic ablation procedure was performed under general anaesthesia with a double-lumen endotracheal tube to facilitate single-lung ventilation. In both centres, the procedure was performed by 2 surgeons. Transoesophageal echocardiography was used to exclude the presence of a left atrial thrombus. Utilizing 3 bilateral ports (12, 12 and 5 mm) with 8–10mmHg CO_2_ insufflation, the pericardium was opened anterior to the phrenic nerve on the right side. After dissecting the pericardial reflection, the superior and inferior guides of the Cardioblate Gemini-S (Medtronic, Minneapolis, MN, USA) were introduced through the transverse and oblique sinuses. On the left, the pericardium was opened posterior to the phrenic nerve, where the guides were retrieved and subsequently attached to the bipolar irrigated radiofrequency clamp. The clamp was then positioned to encircle the left PVs and left atrial posterior wall as depicted in Fig. 1. Ablation was performed for a minimum of 4 times with the convexity of the clamp facing towards the atrial myocardium to apply left PV antrum lesions, and for a minimum of 4 times with the concavity of the clamp facing towards the myocardium to create the roof- and inferior lesions. For each application, energy was delivered until impedance measurements indicated transmurality (based on the generator algorithm). Between applications, the ablation clamp was repositioned to ensure lesion continuity. The same lesions were repeated on the right side to create the right PV antrum lesion and to close the box around the left atrial posterior wall. For a schematic illustration of the lesion set, see Supplementary Material, Fig. S1.

Following left and right lesion application, electrical isolation of the PVs and box was tested. Entrance block was defined as the absence of sharp electrograms in each of the PVs and box, when comparing sensed bipolar electrograms to baseline measurements (MAPS device, Medtronic). Electrical cardioversion was performed if sinus rhythm had not been achieved during the procedure. If sinus rhythm was achieved, exit block was defined as absence of conducted atrial activity during pacing at 10 V, 2 ms pulse width in all PVs and the box. Visual confirmation of pacing viable myocardium was ensured to exclude false-positive exit block measurements. If entrance and/or exit block were absent following lesion set completion, a series of additional radiofrequency applications were performed, and entrance/exit block measurements were repeated. The absence of entrance and/or exit block despite additional radiofrequency applications was regarded as failed entrance/exit block.

At the end of the procedure, the left atrial appendage (LAA) was excluded using a 60 mm stapler (Endo GIA Universal Stapler, Medtronic or Powered Echelon, Ethicon, NJ, USA), and was performed based on thromboembolic risk, appendage anatomy and stapling safety, as determined by the surgeon.

AADs were continued perioperatively. Patients not on an AAD received sotalol or amiodarone postoperatively, or in case of a contraindication for these drugs, a beta-blocker was started. AAD was used throughout the 3-month blanking period and discontinued after the first follow-up if the patient was in sinus rhythm. Oral anticoagulation was discontinued 2–3 days before surgery, was resumed until 6 months after the procedure and discontinued based on rhythm and CHA_2_DS_2_-VASc score, at the discretion of the cardiologist.

### Follow-up

The follow-up procedure differed between the 2 centres. In the OLVG, follow-up of the first 3 years was performed based on rhythm monitored through a preoperatively implanted loop recorder (ILR; Reveal XT^®^ or Linq^®^, Medtronic), with an additional 24-h Holter performed at 5 years. In the LUMC, standard follow-up consisted of 24-h Holter monitoring at 3 and 6 months, 1, 2 and 5 years. Failure was defined as any episode of AF, atrial flutter or atrial tachycardia lasting ≥30 s on 24-h Holter, electrocardiogram or ILR/pacemaker interrogation after a 3-month blanking period. Patients were asked to visit the hospital in case of symptoms suggestive of arrhythmia for additional electrocardiogram and/or 24-h Holter monitoring. Patients with suspected reconnection due to the presence of frequent symptomatic AF/atrial flutter/atrial tachycardia recurrences were invited for an endocardial electrophysiological evaluation and ablation.

### Statistical analysis

Statistical analysis was performed using SPSS (v25, IBM Corporation, NY, USA) and graphs were plotted in GraphPad Prism (v8.1, GraphPad Software Inc., CA, USA). Categorical data are presented as counts and percentages, whereas numerical data are expressed as mean (standard deviation) for normally distributed data or as median [interquartile range] for non-normally distributed data. The normality of the data was assessed using the Shapiro–Wilk test. Numeric data were compared using the independent sample *t*-test or Mann–Whitney *U* test (for normally and non-normally distributed data, respectively), and categorical data were compared using the chi-squared test or Fisher’s exact test in case of low expected counts. Arrhythmia burden between baseline and 2-year follow-up was compared using a paired samples *t*-test.

Freedom from first atrial arrhythmia recurrence was calculated using the Kaplan–Meier method. Graphs were truncated at 5 years to allow for sufficient numbers of patients at risk. Thoracoscopic-only freedom was the freedom from first atrial arrhythmia recurrence following thoracoscopic ablation only after the blanking period. Thoracoscopic + endocardial touch-up freedom was calculated from the last performed ablation procedure, being either the thoracoscopic procedure or an endocardial touch-up intervention. Predictors of atrial arrhythmia recurrence following thoracoscopic ablation were identified using multivariable Cox regression analysis. Variates with a *P*-value smaller than 0.20 in the univariate analysis were included in the multivariable model. Hazard rates were calculated using Cox regression analysis. Due to the non-proportional hazards when comparing continuous and intermittent monitoring, landmark analysis was used to compare recurrence rates from baseline to medium-term (2 years) follow-up and from medium-term to long-term (5 years) follow-up. A *P*-value <0.05 was considered significant.

### Ethics statement

This study was conducted with the approval of both local institutional review boards (LUMC: G17.101-10/09/18; OLVG: WO19.105-18/09/19).

## RESULTS

### Baseline demographics

Eighty patients with LSPAF underwent thoracoscopic ablation between 2009 and 2017. Seventy-six patients had a rhythm follow-up longer than 3 months and were included. One patient who died during the blanking period was included as an ablation failure. As a result, 77 patients were analysed in this study (flowchart in Supplementary Material, Fig. S2).

Mean age was 58.9 (7.7) years, 22.1% was female and median time since first AF diagnosis was 3.8 (1.9–6.3) years. The median left atrial volume index was 46 (13) ml/m^2^ and 16% of patients had a history of prior catheter ablation. For an overview of baseline characteristics, see Table 1.

**Table 1:** Baseline clinical characteristics (*n = *77)

Characteristic	Value
Age (years)	58.9 (7.7)
Female	17 (22%)
Time since first AF diagnosis (years)	3.8 [1.9–6.3]
Left atrial volume index (ml/m^2^)	46 (13)
CHA_2_DS_2_-VASc score	1 [0–2]
0	24 (31%)
1	23 (30%)
≥2	30 (39%)
Body mass index (kg/m^2^)	27.5 (3.7)
Moderate mitral insufficiency	11 (14%)
Coronary artery disease	7 (9%)
Hypertension	39 (51%)
Diabetes mellitus	2 (3%)
Prior stroke/TIA	8 (10%)
Preoperative anticoagulation	75 (97%)
Preoperative anti-arrhythmics	52 (68%)
Preoperative pacemaker/ICD	5 (7%)
Continuous rhythm monitoring	35 (46%)
Prior catheter ablation	12 (16%)
Pulmonary vein isolation	9 (12%)
AFL ablation	3 (4%)

Data are presented as *n* (%), mean (SD) or median [IQR].

AF: atrial fibrillation; AFL: atrial flutter; ICD: implantable cardiac defibrillator; IQR: interquartile range; TIA: transient ischaemic attack; SD: standard deviation.

### Perioperative data

The mean procedure time was 184 (60) min. In both centres, a downwards trend in procedure time indicative of a learning curve was observed over the course of the study (Supplementary Material, Fig. S3). The entrance block of the PVs and box was achieved in all patients. Following ablation, 32.5% of patients spontaneously converted to sinus rhythm. After electrical cardioversion in the remaining patients, 88.3% of patients were in sinus rhythm at the end of the procedure. Exit block of the PV and box compartments was confirmed in all patients, which were in sinus rhythm. The LAA was excluded in 66.2% of patients.

### Safety

No conversion to median sternotomy was required. Video-assisted thoracoscopic reoperation was performed due to haemothorax in 5 patients and due to pericardial effusion/tamponade in 1 patient. In addition, transient phrenic nerve paresis with function recovery occurred in 2 patients and pacemaker implantation for sick sinus syndrome was performed in 2 patients. One patient with a contraindication to anticoagulation (and CHA2DS2-VASc of 3) had a cerebrovascular event at Day 9 after surgery and died 54 days after surgery.

### Long-term follow-up

**Figure 2:**
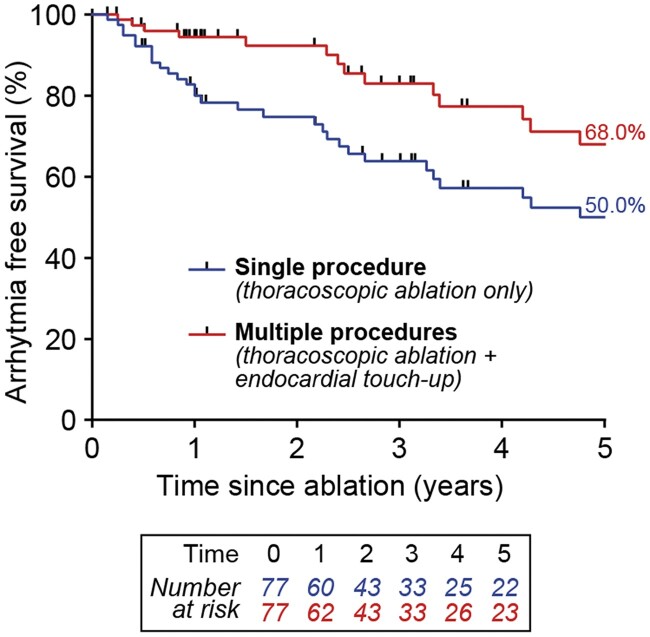
Freedom from all atrial arrhythmias after a single thoracoscopic ablation procedure only (single procedure freedom, blue line) or when allowing catheter touch-up procedures if required (multiple procedure freedom, measured from last procedure, red line).

The median follow-up duration was 3.0 (1.3–5.2) years. Freedom from atrial arrhythmias lasting ≥30 s following thoracoscopic ablation using the Kaplan–Meier method was 74.7% at 2 years [95% confidence interval (CI), 62.7–83.4] and 50.0% at 5 years (95% CI, 36.0–62.6). Catheter touch-up interventions due to symptomatic recurrences were performed in 20.8% of patients. Freedom from any atrial arrhythmia including catheter touch-up interventions was 92.3% at 2 years (95% CI, 82.1–96.8) and 68.0% at 5 years (95% CI, 50.9–80.2) following the last ablation procedure (Fig. 2).

**Figure 3:**
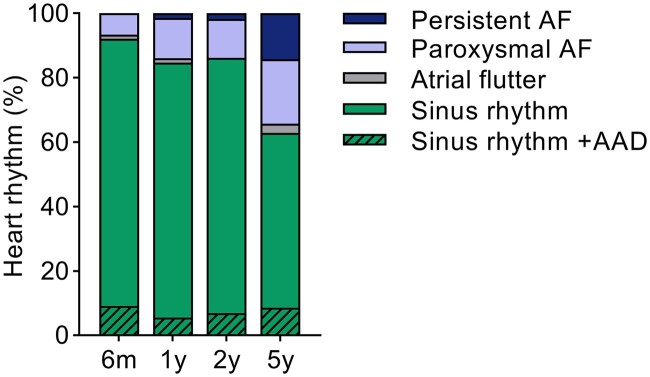
Rhythm status at 6 months and 1, 2 and 5 years following thoracoscopic ablation, including touch-up procedures and antiarrhythmic drug use. Patients at each timepoint are *n = *76, *n = *72, *n = *57 and *n = *35, respectively. AAD: antiarrhythmic drug; AF: atrial fibrillation.

Actual rhythm status measured at the standard follow-up moments revealed sinus rhythm rates of 92.1%, 84.7%, 86.2% and 62.9% at 6 months, 1, 2 and 5 years, respectively. At 5-year follow-up, in addition to 62.9% of patients being in sinus rhythm, 20.0% was in paroxysmal AF, 14.3% in persistent AF and 2.9% was experiencing atrial flutter. Sinus rhythm rates without use of AADs were 82.9%, 79.1%, 79.3% and 54.3% at 6 months, 1, 2 and 5 years, respectively (Fig. 3).

AAD use decreased significantly following thoracoscopic ablation. Preoperatively, 49.4% of patients were taking AADs, compared to 11.8%, 8.3%, 12.1% and 14.3% at 6 months, 1, 2 and 5 years, respectively (all *P *< 0.001 compared to preoperative AAD use). In particular, significant de-escalation of Amiodarone use was seen during follow-up (Table 2).

**Table 2:** Antiarrhythmic drug use during follow-up

	Preoperative (*n = *77)	6 months (*n = *76)	1 year (*n = *72)	2 years (*n = *58)	5 years (*n = *35)
Total AAD use (%)	49.4	11.8^‡^	8.3^‡^	12.1^‡^	14.3^‡^
Flecainide	10.4	7.9	5.5	6.9	8.6
Sotalol	19.5	1.3^‡^	2.8^†^	3.4^†^	5.7
Amiodarone Sotalol	19.5	2.6^†^	–^‡^	1.7^†^	–^†^

*P*-value:

† <0.01,

‡ <0.001 (versus preoperative).

AAD: antiarrhythmic drug.

### Catheter touch-up procedures

Catheter touch-up procedures were performed in 16 (20.8%) of 77 patients. The mean time to first touch-up procedure was 2.0 (1.0) years. Three patients required a second catheter touch-up procedure after the first. Re-isolation of gaps found in the PV- and/or box lesions was performed in 7 patients. In the other 9 patients undergoing a touch-up procedure, the PV and box lesions were already durably isolated. Additional complex fractionated atrial electrograms ablation was performed in 5 patients. Ablation at the base of the stapled LAA was performed in 3 patients, whereas electrical isolation of the non-stapled LAA was performed in 2 patients. A cavo-tricuspid isthmus line was performed in 7 patients and a mitral isthmus line in 8 patients. For an overview of all touch-up procedures, see Supplementary Material, Table S1.

### Predictors of recurrence

Cox regression analysis of relevant clinical factors was performed to assess their impact on atrial arrhythmia recurrence. Multivariable analysis revealed left atrial volume index to be the only independent risk factor for first atrial arrhythmia recurrence following thoracoscopic ablation (hazard ratio: 1.05 for each ml/m^2^ increase in left atrial volume index, 95% CI: 1.02–1.09, *P *= 0.001), as shown in Supplementary Material, Tables S2 and S3.

### Continuous versus intermittent rhythm monitoring

**Figure 4:**
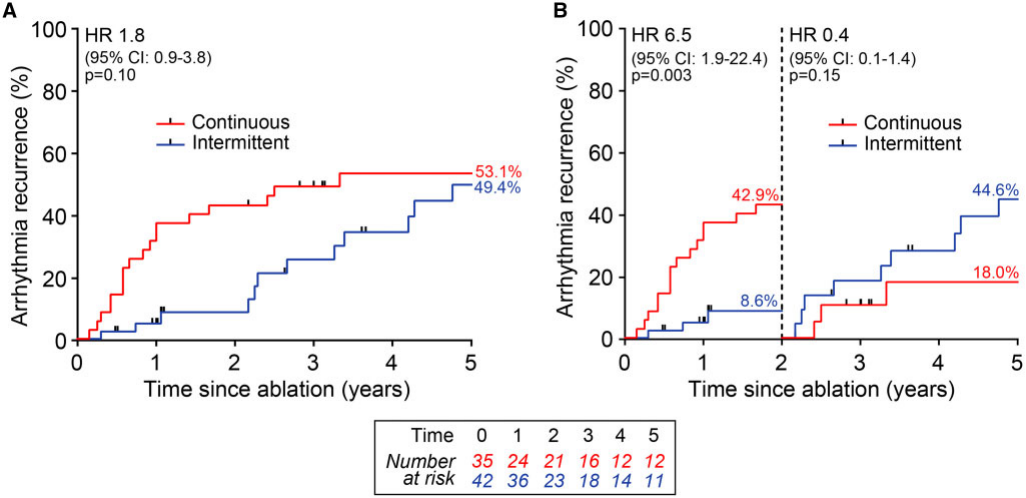
(**A**) Atrial arrhythmia recurrence rates after the thoracoscopic ablation procedure only, in intermittent (blue line) and continuous (red line) rhythm monitoring groups. (**B**) Landmark analysis of recurrence rates from baseline to medium-term (2 years) follow-up and from medium-term to long-term (5 years) follow-up between continuous and intermittent rhythm monitoring. CI: confidence interval; HR: hazard ratio.

Implantable devices capable of continuous atrial rhythm sensing and detection of atrial arrhythmia episodes were present in 45.5% of patients (continuous group). The rest of the cohort relied on 24-h Holter recordings at various timepoints and presentation of symptomatic patients (intermittent group). Here, 91.7% of detected recurrences were due to patients presentation outside standard follow-up moments. Comparison of atrial arrhythmia recurrences between the 2 groups revealed similar recurrence rates at 5-year follow-up (Fig. 4A). However, landmark analysis at medium-term follow-up (2 years) revealed significantly higher recurrence rates in the continuous rhythm monitoring group in the first 2 years (Fig. 4B).

### Arrhythmia burden

**Figure 5:**
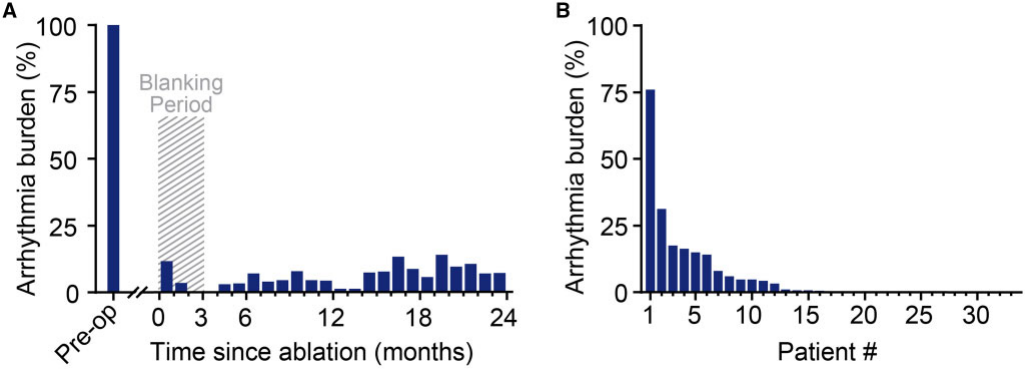
Atrial arrhythmia burden determined using continuous rhythm monitoring devices over the first 2 years following thoracoscopic ablation. (**A**) Changes in the average population burden following ablation. (**B**) Mean burden over the first 2 years following ablation per individual patient, ranked from highest to lowest.

In the month prior to thoracoscopic ablation, mean atrial arrhythmia burden was 100% (*n *= 35). Following thoracoscopic ablation, the mean burden dropped to 0.1% at the end of the 3-month blanking period (Fig. 5A). Over the first year, excluding the blanking period, the atrial arrhythmia burden was 4.2% on average. Two years following ablation, the average atrial burden decreased to 8.0% (*P *< 0.001). During the 2 years following ablation, 48.5% of patients had an average burden <0.1% (Fig. 5B). For an overview of individual patient burden development, see Supplementary Material, Fig S4.

## DISCUSSION

This present study is the first to report long-term efficacy data of thoracoscopic ablation in an LSPAF cohort. Rhythm outcomes were studied for 77 LSPAF patients that underwent thoracoscopic ablation of the PVs and box using a clamping ablation device. The 5-year success rate (defined as freedom from any atrial arrhythmia ≥30 s) was 50% following a single thoracoscopic procedure and was 68% allowing endocardial touch-up procedures (performed in 20.8% of patients). The mean atrial arrhythmia burden was strongly reduced from 100% before ablation, to 0.1% at the end of the blanking period and 8.0% in the second year of follow-up. Comparison of recurrence rates between continuous and intermittent rhythm monitoring groups revealed similar recurrence rates at latest follow-up, but with earlier recurrence detection in patients with continuous rhythm monitoring.

Long-term rhythm control of LSPAF has been challenging to achieve in clinical practice, with electrical cardioversion and AAD therapy being poorly effective in this population [11]. Catheter ablation targeting the arrhythmogenic substrate is frequently performed; however, long-term outcomes remain suboptimal. Tilz *et al.* [1] previously reported 20% freedom from atrial arrhythmias after 5 years following a single catheter ablation procedure, whereas freedom from AF was 38% after 1 or 2 procedures as compared to 45% after multiple (up to 5) procedures. Teunissen *et al.* [4] reported similar rates at 5-year follow-up, being 24% freedom following a single ablation procedure and 41% freedom following multiple procedures.

Conversely, surgical ablation using the Cox-maze IV technique, widely considered the gold standard for AF ablation, has shown high rates of long-term freedom in the LSPAF population. In the recent study of Lapenna *et al*. [12], 67% of LSPAF patients remained free from recurrences 7 years following a stand-alone Cox-maze IV procedure. The group of Ad *et al.* [13] reported comparable rates at 5 years, with 59% of patients being free from atrial arrhythmia recurrences following a single stand-alone minimal access Cox-maze IV procedure. Nonetheless, despite being very efficacious, the invasiveness, required expertise and need for cardiopulmonary bypass of the stand-alone Cox-maze limits widespread applicability.

For surgical ablation to serve as an alternative to catheter ablation, minimal invasiveness, ease of performing the procedure and high efficacy are key. With the advent of thoracoscopic ablation techniques, minimally invasive ‘beating heart’ ablation using simple lesion sets has become commonplace over the past decade. In terms of efficacy, we show in this study for the first time that thoracoscopic ablation is effective in long-term sinus rhythm restoration in LSPAF, as demonstrated by the 68% freedom at 5 years (compared to 38–45% after multiple catheter ablation procedures and 59–67% after stand-alone Cox IV Maze). Particularly, we believe thoracoscopic ablation is effective in the LSPAF population when complemented by catheter touch-up procedures in patients with early recurrence, resulting in a 5-year success rate of 68% after 1 thoracoscopic procedure and a touch-up procedure in 21% of patients.

Literature on totally thoracoscopic ablation efficacy in LSPAF has thus far been limited to short- to medium-term follow-up studies. Haldar *et al.* [14] in the CASA-AF study reported 26% of patients being free from recurrence at 1-year follow-up, whereas Ohtsuka *et al.* [15] reported 47% freedom at 2-year follow-up and van Laar *et al.* [16] reported 54% freedom at 1.7-year follow-up. Although comparison of data between studies is difficult due to differences in patient characteristics and rhythm follow-up, the success rates we report at medium term (75% freedom at 2 years following thoracoscopic ablation only) appear to be higher than previously presented. Here, we believe differences in ablation technique might play a role, as the majority of previous studies have used non-clamping devices to electrically isolate the left atrial posterior wall. We previously reported [8] on the importance of creating the complete lesion set using clamping ablation devices as opposed to non-clamping devices, as the latter are known to deliver inferior lesion transmurality. To confirm whether a completely clamping lesion set is indeed the preferred method, large long-term comparative follow-up studies are needed.

Monitoring of the atrial rhythm following AF ablation has predominantly been performed by intermittent 24-h or 48-h Holter recordings in clinical practice. Despite a trend in recent years towards longer recordings, ILRs have mostly been used in a research setting, where their high temporal data resolution allows for an accurate quantification of the arrhythmia burden. An interesting observation in this study is the notable decrease of an average 100% preoperative arrhythmia burden towards a 0.1% arrhythmia burden measured at the end of the blanking period. This suggests that the thoracoscopic substrate modification (with brief AAD therapy) is sufficient to, at least initially, convert all patients to a situation where they are nearly AF free. In that case, more extensive lesion sets or even hybrid procedures would not be needed to further improve initial success, but rather to prevent late recurrences. With 50% of the patients remaining free from recurrences at long-term follow-up after a single procedure, an initial strategy of a more aggressive ablation would not be the preferred approach, as it would result in overtreatment of a large percentage of patients. Still, the reported strategy of the current study may already overtreat patients, as 20–24% pulmonary vein isolation-alone responders have been reported in endocardial ablation studies [1, 4].

Furthermore, another interesting observation of continuous rhythm monitoring is the earlier, but not more numerous documentation of recurrences. Since the intermittent group mostly relies on the presentation of patients with symptomatic recurrences (92% of recurrences were based on patients presenting outside standard follow-up moments), this would indicate that asymptomatic AF episodes as detected by continuous monitoring precede the symptomatic AF in these patients. Whether use of continuous rhythm monitoring would provide additional value in a clinical setting remains to be determined.

Although there are no large registries for minimally invasive surgical ablation that provide robust insight into the incidence of adverse events, data from smaller studies [16–18] show complication rates in the range of 7.8–23.0% per procedure (for reference, 14.3% in this study). This is higher than the complication rates for catheter ablation procedures which have been well studied and reported in worldwide surveys, and generally range between 3.9% and 12.6% [1, 4, 19, 20]. However, the treatment of patients with symptomatic LSPAF using endocardial techniques generally requires multiple procedures and thus the risks are cumulative. Therefore, the optimal balance between the number of procedures, efficacy and risks in the treatment of these patients needs to be determined. The data reported in this study show that thoracoscopic ablation may play a role in the treatment strategy of patients with LSPAF. Multicentre registries with larger patient groups, preferably including quality of life measurements, are needed to answer the question whether the strong arrhythmia outcome is worth the cost in procedural complications. Finally, cardiologists and surgeons should discuss with their patients all pros and cons of a more invasive surgical procedure with an anticipated superior efficacy, but with a higher event rate, to achieve optimal shared decision-making.

### Study limitations

The retrospective nature and small number of patients in this study influence the conclusions that can be drawn from the data. Actual AF freedom rates might be overestimated in this study as asymptomatic arrhythmia episodes may not have been detected by intermittent 24-h Holter monitoring. Particularly between 2 and 5 years, no systematic Holter monitoring was performed and detection of recurrence was mainly based on patients presenting to their physician. Due to limited battery capacity of ILRs, rhythm monitoring in the continuous group beyond 3 years relied on incidental 24-h Holter monitoring. The comparison of continuous and intermittent follow-up in relation to rhythm outcome may be limited by the majority of the patients in the continuous group (80%) being derived from 1 centre, possibly influencing outcomes by differences in clinical baseline characteristics and risk factors, and/or clinical protocols and practice. A comparison of baseline, procedural and follow-up characteristics between the 2 groups has been provided in Supplementary Material, Table S4.

## CONCLUSION

Thoracoscopic PV and box isolation is effective in long-term restoration of sinus rhythm in LSPAF, especially when the thoracoscopic procedure is complemented by an endocardial touch-up procedure in case of recurrence, as demonstrated by the 68% freedom rate at 5 years. Continuous rhythm monitoring revealed earlier, but not more numerous documentation of recurrences up to long-term follow-up.

## SUPPLEMENTARY MATERIAL

Supplementary material is available at *ICVTS* online.

## Funding

The Department of Cardiothoracic Surgery, LUMC has previously received an unrestricted research grant from Medtronic.

**Conflict of interest:** The authors have no disclosures. 

## Author contributions

**Niels Harlaar:** Conceptualization; Data curation; Formal analysis; Writing—original draft; Writing—review & editing. **Maurice A. Oudeman:** Data curation; Writing—review & editing. **Serge A. Trines:** Conceptualization; Writing—review & editing. **Gijsbert S. de Ruiter:** Investigation; Writing—review & editing. Bart J. Mertens: Formal analysis. **Muchtair Khan:** Investigation; Writing—review & editing. **Robert J.M. Klautz:** Supervision; Writing—review & editing. **Katja Zeppenfeld:** Investigation; Writing—review & editing. **Andrew Tjon:** Investigation; Writing—review & editing. **Jerry Braun:** Conceptualization; Writing—review & editing. **Thomas J. van Brakel:** Conceptualization; Supervision; Writing—review & editing.

## Reviewer information

Interactive CardioVascular and Thoracic Surgery thanks Heyman Luckraz, Yoshiharu Soga and the other anonymous reviewers for their contribution to the peer review process of this article.

## Supplementary Material

ivab355_Supplementary_Material

## ABBREVIATIONS

AAD: Antiarrhythmic drug
AF: Atrial fibrillation
LAA: Left atrial appendage
LSPAF: Long-standing persistent atrial fibrillation
LUMC: Leiden University Medical Center
ILR: Implantable loop recorder
PV: Pulmonary vein
