# Supplementary material for: Long-term follow-up of thoracoscopic ablation in long-standing persistent atrial fibrillation
Source: Interact Cardiovasc Thorac Surg. 2021 Dec 27;34(6):990–8. doi: 10.1093/icvts/ivab355 (PMC9159446; doi:10.1093/icvts/ivab355)
Supplement: ivab355_Supplementary_Material [file ivab355_supplementary_material.docx]

**Supplementary Material**


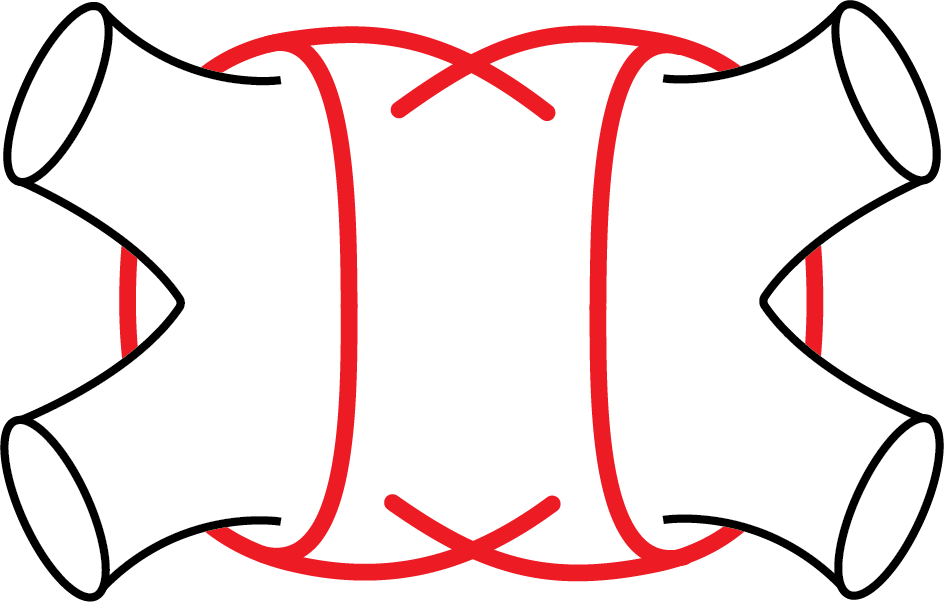
**Supplementary Figure 1:** Schematic of the Gemini-S lesion set used to isolate the pulmonary veins and left atrial posterior wall during thoracoscopic ablation.

**
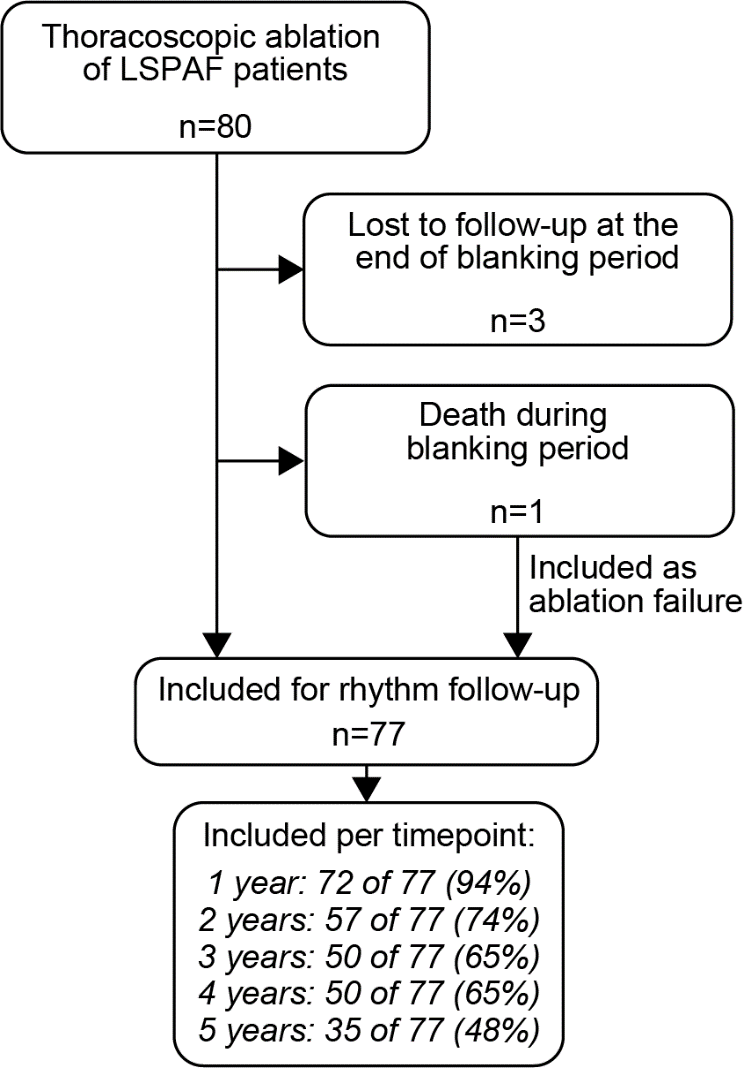
**

**Supplementary Figure 2:** Flowchart depicting the selection of patients for the long-term rhythm analysis following ablation, as well as number included per timepoint.

**
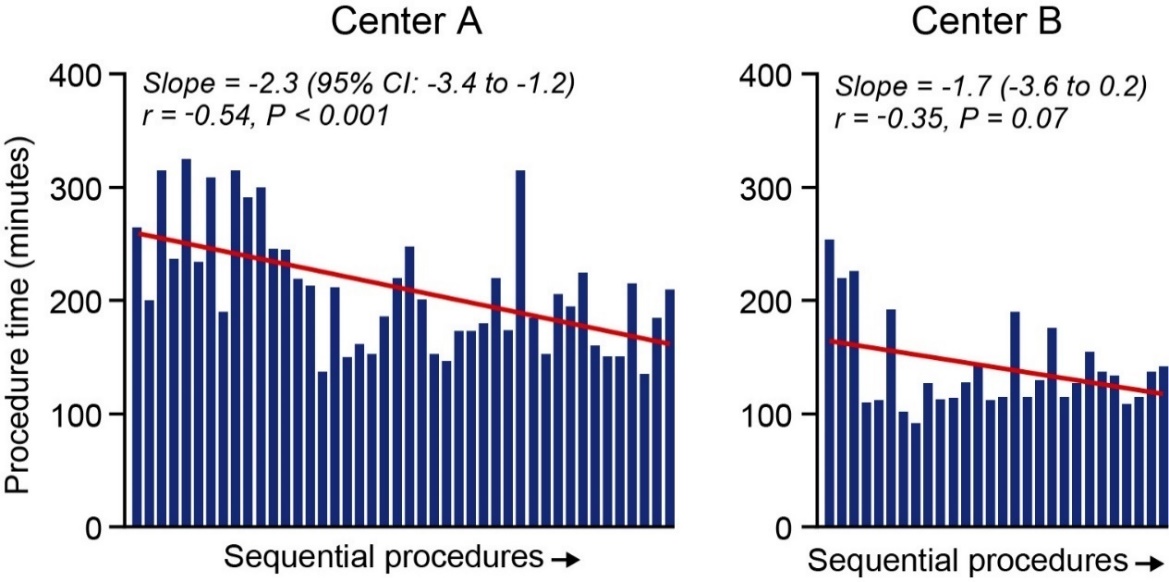
Supplementary Figure 3:** Sequential procedure times plotted of the two centers included in this study with interpolation of a linear standard curve (in red) to assess trend directionality. Respective mean decreases in procedure time of 2.3 and 1.7 minutes per procedure.

**
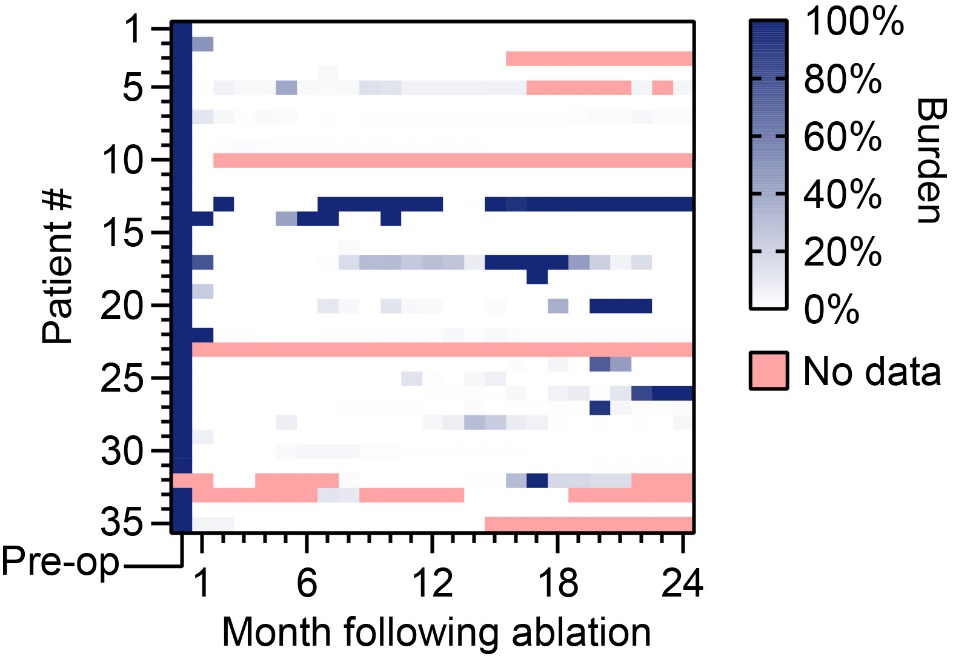
**

**Supplementary Figure 4:** Atrial arrhythmia burden shown per patient over the first two years following thoracoscopic ablation. Each row represents an individual patient.

**Supplementary Table 1:** Details of catheter touch-up interventions following recurrence

| **Patient** | **Type of SVT** | **Interval**  **(months)** | **Touch-up intervention** |
| --- | --- | --- | --- |
| 1^A^ | AF | 6 | Reisolation of LPV/Box + CFAE ablation (LA) + CTI lesion |
| 2^B^ | AF | 10 | MI lesion + CFAE ablation (RAA/IAS) |
| 2^B^ | AFL | 18 | CTI lesion + CFAE ablation (IAS) |
| 3^A^ | AF | 12 | CFAE ablation (LA) + LAA isolation + VCS isolation |
| 4^A^ | AF | 13 | Reisolation of LPV/RPV/Box |
| 5^A^ | AFL | 13 | MI lesion |
| 6^A^ | AFL | 18 | CTI lesion |
| 7^A^ | AF | 19 | Reisolation of LPV/RPV/Box + CTI lesion |
| 7^A^ | AF | 29 | Reisolation of RPV/Box + CTI lesion |
| 8^B^ | AF | 22 | Reisolation of LPV/RPV/Box + CTI and MI ablation |
| 9^B^ | AF | 23 | Reisolation of Box + MI lesion |
| 10^B^ | AF | 27 | LAA isolation + CTI lesion |
| 11^B^ | AF | 28 | Ablation LAA ridge + MI lesion + CS ablation |
| 12^B^ | AF | 32 | Reisolation of Box + LAA base + MI lesion |
| 13^B^ | AF | 35 | Ablation LAA ridge |
| 14^A^ | AF/AT | 36 | Isolation of VCS |
| 14^A^ | AF/AT | 42 | Reisolation of VCS + CFAE ablation (RA) |
| 15^B^ | AF | 46 | CFAE ablation (LA) + MI lesion |
| 16^A^ | AF/AFL | 47 | MI and CTI lesions |
| AF: atrial fibrillation, AFL: atrial flutter, AT: atrial tachycardia, CFAE: complex fractionated atrial electrograms, CS: coronary sinus, CTI: cavotricuspid isthmus, IAS: interatrial septum, LAA: left atrial appendage, LA: left atrium, LPV: left pulmonary veins, MI: mitral isthmus, RPV: right pulmonary veins, RAA: right atrial appendage, RA: right atrium, SVT: supraventricular tachycardia, VCS: vena cava superior  Procedure performed in: ^A^ Center A, ^B^ Center B | | | |

**Supplementary Table 2:** Cox regression analysis for predictors of atrial arrhythmia recurrence.

| **Variable** | **HR (95% CI)** | ***P*-value** |
| --- | --- | --- |
| ***Univariate*** |  |  |
| Age | 1.00 (0.96-1.05) | 0.86 |
| Female gender | 1.32 (0.62-2.85) | 0.47 |
| Years since first AF episode | 1.12 (1.00-1.26) | 0.05 |
| Left atrial volume index | 1.05 (1.02-1.08) | 0.001 |
| Body mass index | 0.98 (0.91-1.07) | 0.72 |
| Prior catheter ablation | 1.99 (0.81-4.88) | 0.13 |
| Sinus rhythm at end of procedure | 0.65 (0.28-1.52) | 0.33 |
| LAA exclusion | 0.71 (0.35-1.44) | 0.34 |
| ***Multivariable*** |  |  |
| Years since first AF episode | 1.08 (0.96-1.22) | 0.18 |
| Left atrial volume index | 1.05 (1.02-1.09) | 0.001 |
| Prior catheter ablation | 1.06 (0.41-2.76) | 0.91 |
| CI: confidence interval, HR: hazard ratio, LAA: left atrial appendage | | |

**Supplementary Table 3:** Cox regression analysis of categorized LAVI as predictor of atrial arrhythmia recurrence to demonstrate linearity.

| **LAVI** | **HR (95% CI)** | ***P*-value** |
| --- | --- | --- |
| <35 ml/m^2^ | Reference group |  |
| 35-45 ml/m^2^ | 8.6 (1.1-66.5) | 0.04 |
| 45-55 ml/m^2^ | 10.3 (1.3-82.6) | 0.03 |
| 55-65 ml/m^2^ | 23.5 (2.8-195.3) | 0.003 |
| >65 ml/m^2^ | 23.9 (2.8-205.6) | 0.004 |
| CI: confidence interval, HR: hazard ratio, LAVI: left atrial volume index | | |

**Supplementary Table 4:** Patient baseline, procedural and follow-up characteristics of the intermittent monitoring group and continuous rhythm monitoring group.

| **Characteristic** | **All**  **(n=77)** | **Intermittent**  **(n=42)** | **Continuous**  **(n=35)** | ***P*-value** |
| --- | --- | --- | --- | --- |
| ***Baseline characteristics*** |  |  |  |  |
| Age (years) | 58.9 (7.7) | 58.9 (7.2) | 58.9 (8.4) | 0.99 |
| Female | 17 (22%) | 28 (80%) | 32 (76%) | 0.69 |
| Time since first AF diagnose (y) | 3.8 [1.9-6.3] | 3.0 [1.5-5.2] | 4.7 [2.9-6.8] | 0.02 |
| Left atrial volume index (ml/m^2^) | 46 (13) | 43 (12) | 50 (13) | 0.02 |
| CHA_2_DS_2_-VASc score | 1 [0-2] | 1 [0-2] | 1 [0-2] | 0.11 |
| Body Mass Index (kg/m^2^) | 27.5 (3.7) | 26.7 (3.4) | 28.5 (3.9) | 0.03 |
| Prior catheter ablation | 12 (16%) | 5 (12%) | 7 (20%) | 0.33 |
| ***Procedure*** |  |  |  |  |
| LAA exclusion | 51 (66%) | 21 (50%) | 30 (86%) | <0.01 |
| Sinus rhythm end of procedure | 68 (88%) | 35 (83%) | 33 (94%) | 0.17 |
| ***Follow-up*** |  |  |  |  |
| Follow-up duration | 3.0 [1.3-5.2] | 2.6 [1.0-5.0] | 3.0 [3.0-5.6] | 0.78 |
| Catheter touch-up procedures | 16 (21%) | 6 (14%) | 10 (29%) | 0.15 |
| Number of touch-up procedures* | 1 [1-1] | 1 [1-2] | 1 [1-1] | 0.26 |
| AAD use at latest follow-up | 5 (14%) | 3 (16%) | 2 (13%) | 0.90 |
| Data are presented as *n* (%), mean (SD) or median [IQR]. *Within individuals requiring a touch-up procedure.  AAD: antiarrhythmic drug, AF: atrial fibrillation, IQR: interquartile range, TIA: transient ischemic attack, SD: standard deviation | | | | |
